# Specific Dysphoric Symptoms Are Predicted by Early Maladaptive Schemas

**DOI:** 10.1155/2014/231965

**Published:** 2014-01-08

**Authors:** Roberta Trincas, Cristina Ottaviani, Alessandro Couyoumdjian, Katia Tenore, Grazia Spitoni, Francesco Mancini

**Affiliations:** ^1^Scuola di Psicoterapia Cognitiva S.r.l., Viale Castro Pretorio 116, 00185 Roma, Italy; ^2^IRCCS Santa Lucia Foundation, Via Ardeatina 306, 00142 Rome, Italy; ^3^Department of Psychology, Sapienza University of Rome, Via dei Marsi 78, 00185 Rome, Italy

## Abstract

Early maladaptive schemas (EMSs) are cognitive patterns resulting from unmet core emotional needs in childhood that have been linked to the development of psychopathology. As depression is a multifaceted phenomenon, we hypothesized that specific dysphoric symptoms would be predicted by different EMSs. Four hundred and fifty-six participants completed a measure of EMSs (Young Schema Questionnaire) and reported on the severity of the symptoms of criterion A for major depression in DSM-IV during the occurrence of a dysphoric episode in the previous 12 months. A series of stepwise multiple regression analyses were performed to investigate the predictive power of the EMSs for the severity of each specific depressive symptom. When controlling for gender and current levels of depression, specific symptoms were predicted by different EMSs: sadness by Negativity/Pessimism; anhedonia by Failure; self-harm by Emotional Deprivation and Vulnerability to Harm or Illness; worthlessness by Failure and Negativity/Pessimism; psychomotor retardation/restlessness by Vulnerability to Harm or Illness and Entitlement/Grandiosity; and poor concentration by Insufficient Self-Control/Self-Discipline. The more physical symptoms of fatigue, insomnia/hypersomnia, and appetite loss/appetite gain were not predicted by any of the EMSs. Although the cross-sectional design of the study does not allow for conclusions about the direction of effects, results suggest that depression is not a unitary phenomenon and provide a possible explanation for previous inconsistent findings.

## 1. Introduction

In recent years, the view that depression is not a unitary phenomenon has received much support and several studies demonstrated the possibility to identify different kinds of depressive reactions associated with specific types of adverse life events [1, 2]. According to cognitive theories of depression [3], it is not the negative event itself but the way the individual interprets the event that leads (or not) to depressive symptoms [4]. As a consequence, when a stressful life event occurs, individuals who have negative cognitive schemas or core beliefs are at an increased risk for depression.

The Young Schema Theory [5] expands on Beck's theory, offering an explanation of the developmental origins of these cognitive factors. According to the theory, individuals who experience toxic events during childhood are prone to develop dysfunctional character traits called “early maladaptive schemas” (EMSs). Young et al. [5] identified 18 EMSs and developed an ad hoc instrument for their assessment: the Young Schema Questionnaire [6]. To date, this self-report measure has been mainly used to study the relationship between EMSs and personality disorders. In the past 15 years, however, the schema model has been also shown to be relevant to mood disorders [7]. Studies have been conducted on both clinical and nonclinical samples, showing that people with mood disorders tend to have higher scores on most or all EMSs compared to healthy controls [8]. In general, findings suggest that EMSs are underlying character traits that remain stable over time rather than a simple reflection of symptoms [9, 10]. For example, history of depression is associated with higher EMSs even in the absence of current depressive symptoms [11], and depressed mood induction in a nonclinical sample had no significant effect on EMSs [12].

The few studies relating specific schema domains and depression, however, found a wide and inconsistent range of EMSs [7]. For example, Calvete and colleagues [13] found that the EMSs *failure*,* defectiveness/shame*, *and self-sacrifice* were associated with depressive symptom severity in non-clinically depressed samples. Another study showed that the EMSs *Defectiveness/Shame*,* Insufficient Self-Control*,* Vulnerability*, *and Incompetence/Inferiority* were cross-sectionally related to depressive symptom severity in undergraduate students [14]. Schmidt and colleagues [15] found that the original *Dependency and Defectiveness/Shame *EMSs accounted for 33% of the variance of depression in a nonclinical sample. Calvete et al. [13] reported that the *Defectiveness/Shame, Insufficient Self-Control, Incompetence, and Vulnerability to Harm or Illness* EMSs explained 63% of the variance of self-reported depressive symptoms while in Stopa et al. [16] the EMSs *Abandonment, Defectiveness/Shame, Subjugation, and Self-Sacrifice* explained 43% of the variance of depression. In Welburn and colleagues [17], 12 of 15 assessed EMSs were significantly correlated with the symptoms of depression, but only the *Abandonment and Insufficient Self-Control* EMSs made a unique contribution to the prediction of depression.

A possible explanation for such inconsistencies comes from the use of different samples, such as groups with a wide range of comorbidities, mixed clinical samples (mood and anxiety disorders), and nonclinical groups [9, 13–17]. In fact, when samples with different psychopathological conditions (e.g., chronic and nonchronic depression and bulimia nervosa) were compared, results showed differences in their EMSs [18, 19].

Here, we propose that another possible explanation for such contradictory findings may derive from the simplistic view of depression as a unitary phenomenon. In line with a dimensional perspective of psychopathology that postulates the existence of a continuum from low mood to clinical depression, we investigated dysphoric reactions after a stressful episode in healthy individuals [20]. We specifically hypothesized that certain EMSs would be associated with different symptoms of depression. According to cognitive theory [21], we assumed that when a stressful life event occurs, specific schemas are activated that affect the way the individual interprets the event, leading to specific depressive symptoms.

## 2. Methods

### 2.1. Participants

The data are part of a larger study investigating cognitive factors in dysphoric episodes [4]. The study was conducted through an online survey, and participants were recruited via advertisements on the internet. Seven hundred and eighty-nine participants agreed to participate and 511 completed the study (64.8%). In all, 456 (89.2%) reported the occurrence of a dysphoric episode (i.e., a 5-day period of low mood) during the previous 12 months. The sample consisted of 328 women (mean age = 38.8.1 (1.5) years) and 128 men (mean age = 42.7 (9.7) years). All subjects were Caucasians. Fifty-six subjects (12.3%) had a past diagnosis of depression, 80 (17.5%) took antidepressants in the past, and 36 (7.9%) were taking psychiatric medications other than antidepressants at the time of the study. The majority of the participants were in paid employment (*n* = 397) and had completed a higher vocational or university education (*n* = 364).

### 2.2. Procedure

The survey was administered in a single session by Questionpro.com, which guarantees the privacy and confidentiality of the respondents, and it took about 45 min to complete. As the online administration provided us with a measure of the time taken to fill out the questionnaire, we were able to exclude outliers (less than 2% of the respondents). After providing instructions and informed consent, all respondents completed a series of forms in the same order as presented below. After survey completion, participants were debriefed and thanked for their time. Debriefing consisted of a brief explanation of the background and aim of the study and feedback of subjects' personal scores at standardized questionnaires. The study was conducted according to the Declaration of Helsinki guidelines.

### 2.3. Sociodemographic and Personal Information

After providing instructions and informed consent, participants were asked to complete a sociodemographic form that included items regarding age, gender, education, and employment.

### 2.4. Questionnaires

Following the methodology described in Keller et al. [1], we assessed the occurrence of the worst dysphoric episode over the previous year, asking subjects to indicate one or more symptoms experienced in the past year. To do so, we presented a list of twelve symptoms extracted from criterion A for major depression in DSM-IV (sadness, anhedonia, fatigue, psychomotor retardation/restlessness, insomnia/hypersomnia, appetite loss/appetite gain, self-harm, poor concentration, and worthlessness) for at least 5 days. Participants were asked to signal how much each symptom interfered with their daily life (from 0, absence of the symptom, to 4, symptom interfering completely with daily life). If they did not experience a dysphoric episode in the previous year (i.e., they scored 0 to all symptoms), they automatically exited the survey. Then, we asked if something happened that might have contributed to make them feel that way, and possible answers were (a) no, I cannot identify any particular event and (b) yes, the symptoms are the consequences of particular events. When the answer was negative, participants were associated with the “no specific cause” category; when it was affirmative, they were asked to further identify the plausible reason for this period of low mood (ALE), first describing it in a free-format paragraph and then selecting the single most likely cause from Keller et al. [1] categories (failure, health problems, interpersonal conflict, death of a loved one, romantic breakup, stress, scare, and others).

### 2.5. Young Schema Questionnaire-Short Form 3

The YSQ-S3 [6] is a 90-item questionnaire that assesses 18 EMSs. Each scale consists of five items, and participants are asked to rate the items using a 6-point Likert scale (1 = completely untrue of me, 2 = mostly untrue of me, 3 = slightly more untrue than true, 4 = moderately true of me, 5 = mostly true of me, and 6 = describes me perfectly). The 18 EMSs are (1)* Abandonment, *(2)* Mistrust/Abuse, *(3)* Emotional Deprivation, *(4) *Defectiveness/Shame, *(5)* Social Isolation/Alienation*, (6)* Dependence/Incompetence*, (7)* Vulnerability to Harm or Illness, *(8)* Enmeshment/Undeveloped Self, *(9)* Failure, *(10)* Entitlement/Grandiosity, *(11)* Insufficient Self-Control/Self-Discipline, *(12)* Subjugation, *(13)* Self-Sacrifice, *(14)* Approval-Seeking/Recognition-Seeking, *(15)* Negativity/Pessimism, *(16)* Emotional Inhibition, *(17)* Unrelenting Standards/Hypercriticalness,* and (18)* Punitiveness*. In this study, the average of the item scores was computed for each schema (range = 1 to 6), as this method has been reported and indicated as the most reliable in discriminating between clinical and nonclinical groups [19, 22].

### 2.6. Center for Epidemiologic Studies Depression Scale

To assess current depressive state, we administered the Italian version of the Center for Epidemiologic Studies Depression Scale (CES-D) [23] which has shown good reliability and convergent validity with related self-report measures. The CES-D is a 20-item self-report scale that assesses the frequency of occurrence of symptoms of depression during the past week. Total score ranges from 0 to 60. Standard cut-offs are >16 for mild depression and >23 for clinical depression.

### 2.7. Statistical Analysis

All data are expressed as means (SD). Data analyses were performed with Statistica 8 (StatSoft. Inc., USA).

First, the intercorrelations between the different EMSs were computed. To investigate the predictive power of the YSQ dimensions for the occurrence of specific depressive symptoms during a past dysphoric episode, nine forward stepwise multiple regression analyses were performed, with each symptom (sadness, anhedonia, fatigue, psychomotor retardation/restlessness, insomnia/hypersomnia, appetite loss/appetite gain, self-harm, poor concentration, and worthlessness) as the dependent variable. Due to the number of EMSs and hence comparisons, the *P*-to-enter was Bonferroni adjusted (*P* = 0.0025–0.05/20 comparisons). To control for gender and current level of depression (CES-D score), these variables were always entered as predictors in the models.

## 3. Results

Table 1 shows means and standard deviations for the variables of interest. The average time from the event was 7.3 (4.5) months.  Table 2 shows means and standard deviations for each EMS in men and women. Men had higher levels of *Social Isolation/Alienation* (*P* = 0.01) and *Emotional Inhibition* (*P* = 0.002) and women had higher levels of *Enmeshment/Undeveloped Self* (*P* = 0.02) and *Self-Sacrifice* (*P* = 0.004).

Scores of the different EMSs were intercorrelated (see Table 3), except for the absence of correlation between (1) *Self-Sacrifice* and the EMSs *Abandonment, Social Isolation, Defectiveness/Shame, Failure, Dependence/Incompetence, Vulnerability to Harm or Illness, Emotional Inhibition, Insufficient Self-Control/Self-Discipline, Approval-Seeking/Recognition-Seeking, Negativity/Pessimism*, and (2) *Entitlement/Grandiosity* and *Failure*. In regard to multicollinearity, the present data showed no minimal tolerance problem for the predictors.

Results from the stepwise multiple regression analyses (see Table 4) showed that the current level of depression was significantly associated with all the symptoms experienced at the time of the dysphoric episode. In line with our hypothesis, different EMSs were related to specific symptoms. The symptom of sadness was significantly predicted by *Negativity/Pessimism* (*R* ^2^ = 0.38; *P* < 0.0001); anhedonia was predicted by *Failure* (*R* ^2^ = 0.42; *P* < 0.0001); self-harm was predicted by *Emotional Deprivation* and *Vulnerability to Harm or Illness* (*R* ^2^ = 0.37; *P* < 0.0001); worthlessness was predicted by *Failure* and *Negativity/Pessimism* (*R* ^2^ = 0.45; *P* < 0.0001); Psychomotor retardation/Restlessness was predicted by *Vulnerability to Harm or Illness* and *Entitlement/Grandiosity* (*R* ^2^ = 0.25; *P* < 0.0001); poor concentration was predicted by *Insufficient Self-Control/Self-Discipline* (*R* ^2^ = 0.40; *P* < 0.0001). Occurrence of the more physical symptoms of fatigue, insomnia/hypersomnia, and appetite loss/appetite gain was only predicted by the CES-D scores (*R* ^2^ = 0.14; *P* < 0.0001 for appetite; *R* ^2^ = 0.24; *P* < 0.0001 for fatigue; *R* ^2^ = 0.24; *P* < 0.0001 for sleep) but not by the EMSs.

## 4. Discussion

In the Diagnostic and Statistical Manual of Mental Disorders, 4th Edition, Text Revision (DSM-IV-TR; 2000), distinct subtypes based on symptomatology and its determinants are taken into account for anxiety disorders, while depression is only classified according to temporal criteria. The present study was theoretically driven by recent findings on the existence of different depressive subtypes in response to certain life events [1, 4, 24, 25]. Specifically, we aimed at exploring if symptoms of depression that emerged during a dysphoric episode after a stressful event could be predicted by stable personality characteristics such as the EMSs. Results supported the hypothesis and provided a possible explanation for previous contradictory findings. Our study differs from previous research for two main aspects: first, we considered the specific symptoms of the depressive symptomatology instead of the categorical diagnosis or the severity of depression and second, we considered dysphoric episodes that occurred after a stressful event. The latter aspect supports the assumptions of the Schema Therapy model [5], implying that EMS may remain relatively latent until activated by stressful life events.

First, results showed that the occurrence of sadness after a stressful event was predicted by the EMS *Negativity/Pessimism*, that is, the pervasive focus on the negative aspects of life. Interestingly, despite the reasonableness of such association, this EMS has never been linked to depression. In our opinion, this is the consequence of a reductive and oversimplified view of depression that merges symptoms that are totally different and not necessarily present together.

Second, the occurrence of anhedonia was predicted by the EMS *Failure*, that is, the belief that one is inadequate compared with others. Accordingly, the diathesis-stress model of depression [26] hypothesizes the existence of sociotropic and autonomic individuals characterized by different depressive reactions: the first more likely to show depressive symptoms following interpersonal loss experiences, the second more vulnerable to events of failure. Our results make a step further suggesting the association between the EMS Failure and a specific depressive symptom such as anhedonia. This finding is also in agreement with Calvete et al. [13], who found that this EMS predicted depressive symptoms among 407 undergraduate students.

Self-harm was predicted by *Emotional Deprivation*, (i.e., the feeling that adequate emotional support is not available) and *Vulnerability to Harm or Illness* (i.e., the belief that catastrophe is impending). *Emotional Deprivation* has been previously associated with depressive symptom severity [27]. Moreover, *Vulnerability to Harm or Illness*, together with *Defectiveness/Shame* and *Insufficient Self-Control,* has been shown to explain 63% of the variance of self-reported depressive symptoms [14]. These results are also consistent with a study in which the EMSs *Emotional Deprivation, Dependence/Incompetence, and Vulnerability to Harm* were able to differentiate between suicidal depressed, non-suicidal-depressed, and nonclinical samples [28]. It has to be noted that the EMS *Vulnerability to Harm or Illness, *together with* Entitlement/Grandiosity*, predicted the occurrence of psychomotor retardation/restlessness. It is intriguing that, EMS *Entitlement/Grandiosity* has been previously considered a signature of bipolar disorder [29].

The symptom of worthlessness was predicted by *Failure* and *Negativity/Pessimism* (i.e., the pervasive focus on negative aspects of life). Again, only *Failure* has been previously associated with depression [13].

Finally, poor concentration was predicted by *Insufficient Self-Control/Self-Discipline*, that is the belief that one cannot restrain emotions or impulses. This association is not surprising if we consider that items are, for example: “When tasks become difficult, I usually cannot persevere and complete them.” Moreover, *Insufficient Self-Control *appeared to be a key EMS specific to the symptoms of depression and to MDD in several studies [11, 13–17, 30, 31].

With regard to gender, the only studies that specifically examined differences between men and women in EMSs have been conducted on clinical populations. In a group of opioid users, Shorey et al. [32] showed that women scored significantly higher than men on three schema domains, including disconnection and rejection, impaired autonomy, and other directedness. In line with this result, we found higher levels of *Enmeshment/Undeveloped Self* and *Self-Sacrifice *in women. Studies on gender differences in mood disorders showed that women tend to be less assertive, more prone to helplessness, and more likely to overvalue relationships as sources of self-worth, which are all risk factors contributing to depression [33].

Considering the promise of Schema Therapy as a treatment approach for personality disorders [34], present data may have important clinical implications for its implementation in the therapy of depressive symptoms. In line with a view of depression as a multifaceted condition, present findings suggest the existence of specific depressive states in people with different EMSs who experienced an adverse life event. It has to be noted that, in most studies, the EMSs predicted about the 11% of the variance of the scores at depression inventories [33]; our predictions of each specific depressive symptom were much more powerful (up to 45%).

Several limitations need to be acknowledged. The major limit is the cross-sectional nature of the study that precludes any conclusions about the direction of effects. Moreover, the dysphoric episode occurred before the compilation of the YSQ. As a fundamental assumption in schema theory is that EMSs are stable, trait-like constructs that are resistant to change [5, 12], also during the occurrence of episodes of depression [9, 10], there is no reason to think that this may have biased the results. Third, problems in data interpretation may arise from the use of retrospective self-reports. With regard to generalizability issues, it has to be noted that the sample was self-selected (i.e., willing to participate in an on-line survey) and consists of a majority of females, but this reflects gender disparities in the prevalence of depressive episodes [35].

Limitations notwithstanding, the current study provides preliminary evidence that specific EMSs may be vulnerability factors for specific depressive symptoms. Future studies on depression in general and on the relationship between EMSs and depression in particular should take into account that several depressive reactions exist based on how individual characteristics, such as EMS, determine the interpretation of adverse life events. Clinical implications are evident if we consider that specific schema domains have been recently shown to predict treatment outcome [27].

## Figures and Tables

**Table 1:** Means and standard deviations for the current level of depression (range 0–57) and the symptoms (range 0–4) at the time of the dysphoric episode.

	*M* (SD)
CES-D	16.1 (11.2)
Sadness	3.1 (1.3)
Anhedonia	2.4 (1.4)
Fatigue	2.7 (1.2)
Psychomotor retardation/Restlessness	1.7 (0.9)
Insomnia/Hypersomnia	2.1 (0.9)
Appetite loss/Appetite gain	1.9 (0.8)
Self-harm	1.9 (1.2)
Poor concentration	2.5 (1.3)
Worthlessness	2.3 (1.3)

**Table 2:** Mean EMS scores.

	Men	Women	*t*
Abandonment	2.1 (1.1)	2.2 (1.1)	1.0
Mistrust/Abuse	2.2 (0.9)	2.2 (1.0)	−0.3
Emotional Deprivation	1.8 (0.9)	1.9 (1.1)	0.9
Defectiveness/Shame	1.6 (0.9)	1.5 (1.0)	−0.6
Social Isolation/Alienation	2.4 (1.2)	2.1 (1.1)	−2.6*
Dependence/Incompetence	1.6 (0.7)	1.5 (0.7)	−1.7
Vulnerability to Harm or Illness	1.6 (0.7)	1.8 (0.9)	1.7
Enmeshment/Undeveloped Self	1.6 (0.7)	1.8 (0.9)	2.3*
Failure	1.7 (0.9)	1.7 (1.0)	0.2
Entitlement/Grandiosity	2.7 (1.0)	2.6 (0.9)	−0.7
Insufficient Self-Control/Self-Discipline	2.4 (1.1)	2.2 (0.9)	−1.9
Subjugation	1.9 (0.9)	1.9 (0.9)	−0.2
Self-Sacrifice	2.8 (0.9)	3.1 (1.1)	2.9*
Approval-Seeking/Recognition-Seeking	2.8 (1.0)	2.6 (1.0)	−1.9
Negativity/Pessimism	2.1 (1.0)	2.1 (1.0)	0.1
Emotional Inhibition	2.7 (1.2)	2.4 (1.1)	−3.1*
Unrelenting Standards/ Hypercriticalness	3.3 (0.9)	3.3 (0.9)	0.1
Punitiveness	2.3 (1.0)	2.2 (1.0)	−0.6

*Note*. *P < 0.05.

**Table 3:** Intercorrelations between EMSs.

	1	2	3	4	5	6	7	8	9	10	11	12	13	14	15	16	17	18
(1) Abandonment	∗																	
(2) Mistrust/Abuse	0.55	∗																
(3) Emotional Deprivation	0.43	0.56	∗															
(4) Defectiveness/Shame	0.55	0.53	0.56	∗														
(5) Social Isolation	0.48	0.54	0.57	0.67	∗													
(6) Dependence	0.51	0.30	0.26	0.56	0.47	∗												
(7) Vulnerability to Harm	0.44	0.42	0.27	0.46	0.40	0.45	∗											
(8) Undeveloped Self	0.38	0.33	0.25	0.27	0.32	0.45	0.36	∗										
(9) Failure	0.43	0.29	0.26	0.56	0.45	0.70	0.44	0.38	∗									
(10) Entitlement/Grandiosity	0.36	0.44	0.34	0.25	0.43	0.24	0.26	0.25	0.12	∗								
(11) Insufficient Self-Control	0.44	0.32	0.28	0.41	0.41	0.55	0.37	0.35	0.52	0.47	∗							
(12) Subjugation	0.58	0.49	0.50	0.58	0.58	0.57	0.48	0.50	0.54	0.36	0.55	∗						
(13) Self-Sacrifice	0.15	0.27	0.20	0.03	0.07	0.04	0.12	0.29	0.06	0.22	0.09	0.22	∗					
(14) Approval-Seeking	0.46	0.35	0.21	0.35	0.30	0.41	0.32	0.30	0.35	0.41	0.48	0.45	0.08	∗				
(15) Negativity/Pessimism	0.58	0.55	0.35	0.53	0.47	0.50	0.76	0.39	0.50	0.32	0.44	0.56	0.13	0.44	∗			
(16) Emotional Inhibition	0.36	0.38	0.35	0.43	0.50	0.29	0.29	0.26	0.30	0.28	0.24	0.45	0.09	0.24	0.32	∗		
(17) Unrelenting Standards	0.23	0.33	0.23	0.28	0.40	0.24	0.34	0.24	0.22	0.41	0.18	0.34	0.32	0.31	0.39	0.35	∗	
(18) Punitiveness	0.35	0.41	0.28	0.44	0.42	0.36	0.46	0.23	0.41	0.26	0.30	0.41	0.19	0.31	0.56	0.29	0.53	∗

**Table 4:** Final steps (*P*-to-enter = 0.0025) of the stepwise regression models of EMSs predicting symptoms scores (*n* = 456).

Dep var	Predictors	*B*	SE	*β*
Sadness	Gender	0.18	0.05	0.12
	CES-D	0.06	0.01	0.50
	Negativity/Pessimism	0.19	0.06	0.15
Anhedonia	CES-D	0.07	0.01	0.55
	Failure	0.23	0.06	0.16
Self-Harm	CES-D	0.03	0.01	0.31
	Emotional Deprivation	0.16	0.05	0.13
	Vulnerability to Harm	0.45	0.06	0.31
Worthlessness	CES-D	0.04	0.01	0.36
	Failure	0.36	0.06	0.25
	Negativity/Pessimism	0.25	0.06	0.20
Psychom/Restlessness	CES-D	0.03	0.01	0.30
	Vulnerability to Harm	0.19	0.05	0.17
	Entitlement/Grandiosity	0.18	0.05	0.17
Poor concentration	CES-D	0.05	0.01	0.47
	Insufficient Self-Control	0.34	0.06	0.25

*Note*. *B*: unstandardized regression coefficient; SE: standard error; *β*: standardized regression coefficient.
